# Eugenol Exhibits Cyclic Nucleotide/VASP-Independent Antiplatelet Activity Associated with Inhibition of Arachidonic Acid-Induced JNK Phosphorylation and Reduces Thrombus Formation as Visualized by Real-Time Intravital Imaging

**DOI:** 10.3390/biomedicines14081800

**Published:** 2026-08-11

**Authors:** Chia-Yuan Hsu, Wei-Chieh Huang, Joen-Rong Sheu, Arief Gunawan Darmanto, Cheng-Ying Hsieh, Chih-Wei Hsia

**Affiliations:** 1 Graduate Institute of Medical Sciences, College of Medicine, Taipei Medical University, Taipei 110, Taiwan; m120107023@tmu.edu.tw (C.-Y.H.); d119110003@tmu.edu.tw (W.-C.H.); sheujr@tmu.edu.tw (J.-R.S.); 2 Department of Pharmacology, School of Medicine, College of Medicine, Taipei Medical University, Taipei 110, Taiwan; 3 School of Medicine, Universitas Ciputra, Surabaya 60219, Indonesia; arief.gunawan@ciputra.ac.id; 4 Department of Medical Research, Taipei Medical University Hospital, Taipei 110, Taiwan

**Keywords:** eugenol, platelet activation, arachidonic acid, JNK, cyclic nucleotide, arterial thrombosis

## Abstract

**Background/Objectives**: Platelets are anucleate cells that play a crucial role in primary hemostasis and arterial thrombosis, contributing to cardiovascular diseases. Eugenol, a bioactive phenolic compound, exhibits vasodilatory, antibacterial, and anticancer properties and inhibits platelet aggregation induced by collagen and arachidonic acid (AA). AA is a critical lipid component of the platelet membrane and a precursor for potent signaling molecules that mediate platelet activation. However, the precise mechanisms through which eugenol modulates AA-stimulated platelet activation remain unclear. **Methods**: Human platelets were pretreated with eugenol and subsequently stimulated with AA. Platelet aggregation, ATP release, intracellular calcium mobilization, and P-selectin expression were measured. JNK, p38 MAPK, ERK, and vasodilator-stimulated phosphoprotein (VASP) phosphorylation were analyzed by Western blotting. SP600125, SQ22536, and ODQ were used to examine the involvement of JNK and cyclic nucleotide signaling pathways. Antithrombotic effects were further evaluated in a mouse mesenteric thrombosis model. **Results**: Eugenol significantly suppressed AA-induced platelet aggregation, ATP release, calcium mobilization, and P-selectin expression, selectively reducing JNK phosphorylation without affecting p38 MAPK or ERK. SP600125 produced similar inhibitory effects. Neither SQ22536 nor ODQ reversed eugenol’s inhibitory effects on platelet aggregation. Furthermore, eugenol did not alter VASP phosphorylation at Ser157 or Ser239. In vivo, real-time intravital imaging showed that eugenol and SP600125 delayed thrombus formation and prolonged occlusion time. **Conclusions**: These findings suggest that eugenol exerts inhibitory effects that may involve modulation of JNK phosphorylation, independent of cyclic nucleotide/VASP pathways in AA-induced platelet activation, highlighting its potential as an antithrombotic agent.

## 1. Introduction

In hemostasis and thrombosis, platelet activation plays a crucial role in maintaining vascular integrity and responding to vascular injury [1]. Various agonists activate platelets, including collagen, thrombin, and arachidonic acid (AA), which bind to their respective receptors and trigger intracellular signaling cascades. Among the multiple biochemical pathways that regulate platelet activation, AA metabolism is a key determinant of platelet function. Upon activation, platelets mobilize AA from membrane phospholipids through the enzymatic action of cytosolic phospholipase A_2_ (cPLA_2_), which hydrolyzes AA-containing phospholipids [2]. Once mobilized, AA is primarily metabolized via the cyclooxygenase (COX) pathway, ultimately creating thromboxane A_2_ (TxA_2_), a potent pro-aggregatory and vasoconstrictive mediator, and the prostaglandins D_2_ (PGD_2_) and E_2_ (PGE_2_) [3]. Dysregulation of the COX pathway is implicated in atherothrombosis, wherein excessive platelet activation contributes to cardiovascular disease [4]. Thus, AA metabolism has emerged as a major therapeutic target in antiplatelet interventions.

In addition to the COX pathway, intracellular signaling cascades such as mitogen-activated protein kinases (MAPKs), including extracellular signal-regulated kinase (ERK), p38 MAPK, and c-Jun N-terminal kinase (JNK), play important roles in platelet activation [5,6]. MAPK activation is associated with agonist-induced AA liberation via cPLA_2_ and contributes to key platelet responses, including granule secretion, thromboxane generation, and integrin α_IIb_β_3_ activation [7,8,9]. In contrast, cyclic nucleotides, including cyclic adenosine monophosphate (cAMP) and cyclic guanosine monophosphate (cGMP), are critical second messengers that regulate platelet inhibition. These molecules activate kinases that phosphorylate key targets, such as vasodilator-stimulated phosphoprotein (VASP), thereby attenuating platelet activation and modulating integrin α_IIb_β_3_ activation [10] and actin filament dynamics [11,12]. Together, these signaling pathways maintain a dynamic balance between platelet activation and inhibition.

Antiplatelet agents such as clopidogrel, aspirin, and integrin α_IIb_β_3_ inhibitors play an essential role in suppressing platelet hyperactivation, thereby reducing the risk of thrombotic cardiovascular events [13]. Despite their therapeutic benefits, these medications are frequently associated with adverse effects; aspirin may lead to gastrointestinal bleeding and ulcers, while clopidogrel has been linked to rare but severe hematologic complications, including aplastic anemia and thrombotic thrombocytopenic purpura [13,14]. These limitations underscore an urgent demand for new antiplatelet therapies that offer enhanced efficacy and safety, ideally minimizing or eliminating drug-induced side effects in the prevention and treatment of cardiovascular diseases.

Eugenol, a phenolic compound derived from *Syzygium aromaticum*, exhibits cardioprotective, neuroprotective, and antibacterial activities [15,16,17,18]. Huang et al. demonstrated that eugenol selectively inhibits collagen- and AA-induced platelet activation but has little effect on thrombin- or U46619-induced responses [19]. Its inhibition of collagen-induced platelet activation has been associated with suppression of the PLCγ2/PKC and cPLA_2_/TXA_2_ pathways. However, the mechanisms underlying its effects on AA-induced platelet activation remain incompletely understood. Therefore, this study investigated whether MAPK and cyclic nucleotide/VASP signaling contribute to the antiplatelet effects of eugenol.

## 2. Materials and Methods

### 2.1. Reagents

Eugenol was obtained from Cayman Chemical (Ann Arbor, MI, USA). Anti-p-JNK, anti-p-ERK, anti-JNK antibodies (Abs), anti-p38 MAPK, and anti-ERK were obtained from Cell Signaling (Beverly, MA, USA). Anti-p-p38 MAPK, anti-p-VASP and anti-VASP Abs were bought from Affinity (Cincinnati, OH, USA) or GeneTex (Irvine, CA, USA). AA, dimethyl sulfoxide (DMSO), luciferin–luciferase, nitroglycerin (NTG), prostaglandin E_1_ (PGE_1_), and SP600125 were purchased from Sigma (St. Louis, MO, USA).

### 2.2. Human Platelet Suspension, Aggregation and ATP Release

Human blood was obtained from 32 healthy volunteers, with sex and gender not included as experimental variables. Blood was anticoagulated with acid–citrate–dextrose solution at a 9:1 ratio, and washed platelets were resuspended in Tyrode’s solution containing 3.5 mg/mL BSA and 1 mM Ca^2+^ [20]. Before AA stimulation, platelets were incubated for 3 min with 0.1% DMSO or eugenol at the indicated concentrations (1–5 μM). Platelet aggregation was recorded using a Lumi-Aggregometer (Payton, Scarborough, OT, Canada). ATP release was determined using a luciferin–luciferase reagent added 3 min before AA stimulation. The absorbance was measured with a spectrometer (Hitachi, Tokyo, Japan).

### 2.3. Cytosolic Calcium Mobilization

To assess cytosolic Ca^2+^ concentration, platelets were preincubated with a solvent control (0.1% DMSO), eugenol (5 μM), or SP600125 (a JNK inhibitor) for 3 min before AA was added. The platelets were then incubated with the calcium indicator Fura 2-AM (5 μM, Eugene, OR, USA). Absorbance was measured with a spectrometer at excitation wavelengths of 340 and 380 nm and an emission wavelength of 500 nm to determine Ca^2+^ concentration [20].

### 2.4. Immunoblotting

Washed platelets (3.6 × 10^8^ cells/mL) were preincubated with either eugenol (5 μM) or the solvent control (0.1% DMSO) for 3 min, after which AA was added to initiate activation. The platelets were then resuspended in lysis buffer (200 μL), and protein from the supernatants (80 μg) was separated through gel electrophoresis. Specific proteins were detected with their respective primary Abs, and the optical density of protein bands was analyzed with a video densitometer. Values were normalized to the total protein of interest to determine relative protein expression.

### 2.5. Confocal Laser Fluorescence Microscopy

Resting or AA-stimulated platelets (60 μM; 3 × 10^7^ cells/mL) were fixed on poly-L-lysine-coated coverslips with 4% paraformaldehyde. After permeabilization and blocking, platelets were incubated with specific primary antibodies overnight, followed by Alexa Fluor 488- or 647-conjugated secondary antibodies (Abcam, Waltham, MA, USA). For P-selectin immunostaining, resting or AA (60 μM)-stimulated platelets were fixed on poly-L-lysine-coated coverslips for 1 h. Following centrifugation, pellets were treated with P-selectin conjugated with fluorescein isothiocyanate for 30 min. Samples were then covered with a coverslip using mounting medium. Confocal images were taken using a microscope with a 100× oil immersion objective.

### 2.6. Surface Expression of P-Selectin by Flow Cytometry

Platelets were incubated with eugenol (5 μM) and FITC-conjugated anti-P-selectin monoclonal antibody (2 μg/mL, BioLegend, San Diego, CA, USA) for 3 min. After incubation, platelet activation was induced by the addition of AA (60 μM). The fluorescence intensity of labeled platelets was then analyzed using a flow cytometer (FACScan, Becton Dickinson, San Jose, CA, USA).

### 2.7. Real-Time Intravital Imaging of Vascular Thrombosis in Mouse Mesenteric Vessels

Male ICR mice (6 weeks old, approximately 25 g) were intraperitoneally injected with DMSO (0.1%) or eugenol (6 and 15 mg/kg) and then intravenously injected with sodium fluorescein (15 μg/kg) through the lateral caudal vein, per the method described by Hsia et al. [21]. Male mice were selected to avoid hormonal fluctuations associated with the estrous cycle, which can affect platelet reactivity and thrombus formation. Intraperitoneal sodium pentobarbital (50 mg/kg) was applied as an anesthetic. Mesenteric arteries were irradiated with light at wavelengths < 520 nm to induce microthrombus formation, and real-time monitoring was used to measure the time to vessel occlusion [22]. After the experiment, mice were euthanized immediately by carbon dioxide inhalation at a displacement rate of 20–30% of the chamber volume/min. Death was confirmed by the absence of spontaneous respiration for more than 2 min and the loss of reflexes.

### 2.8. Statistical Analysis

Data are presented as the mean ± standard error of the mean (SEM). For human platelet experiments, *n* represents the number of independent donors; for animal experiments, *n* denotes the number of individual animals. One-way analysis of variance (ANOVA) and subsequent Student–Newman–Keuls post hoc tests were performed to identify significant differences between experimental groups. Where applicable, exact *p* values and 95% confidence intervals were reported for pairwise mean differences, and effect sizes for overall ANOVA effects were reported. Statistical significance was indicated by *p* < 0.05. All analyses were conducted with SAS software (version 9.2; SAS, Cary, NC, USA).

## 3. Results

### 3.1. Inhibitory Effects of Eugenol on AA-Induced Platelet Aggregation and Granule Secretion

**Figure 1:**
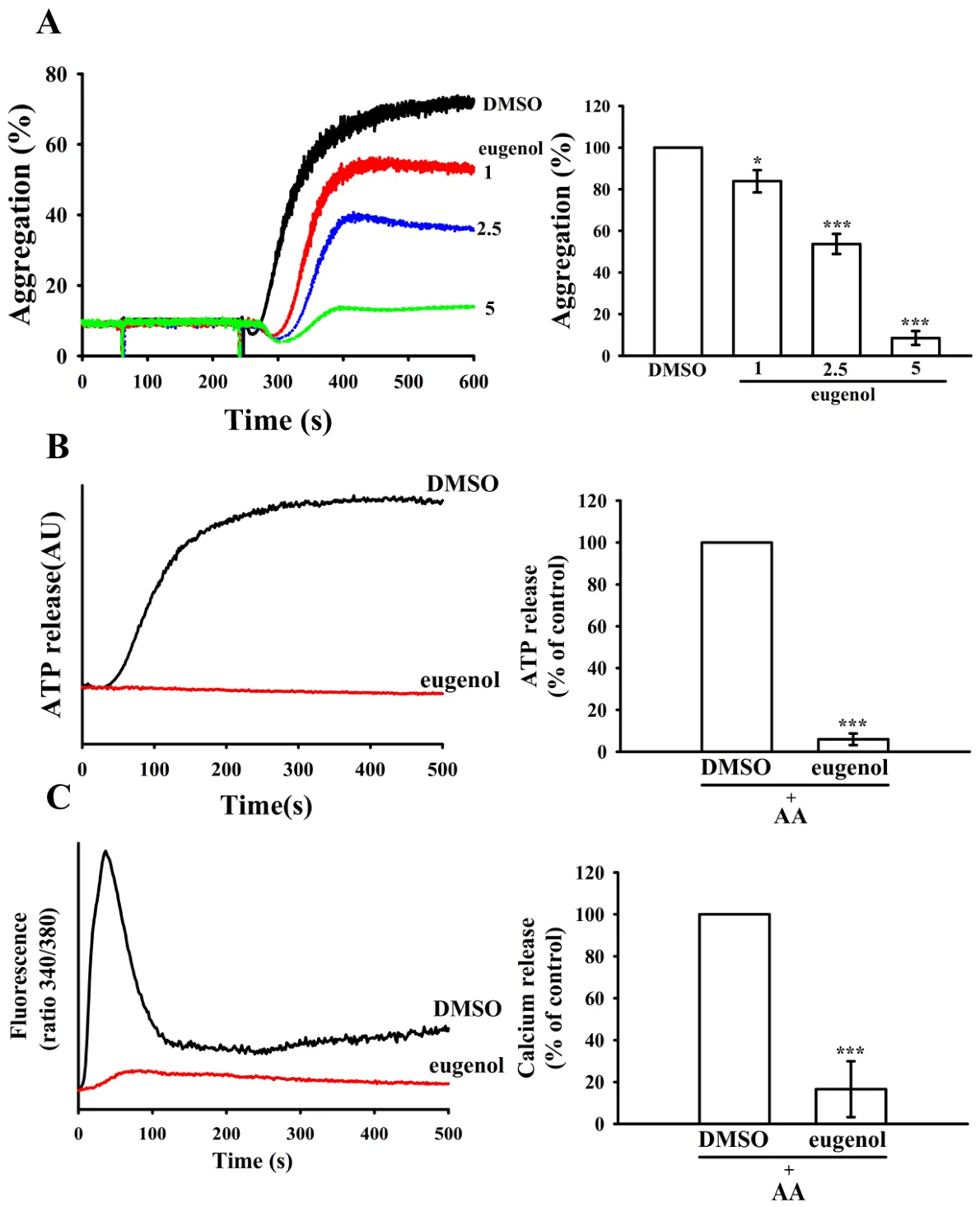
Effects of eugenol on AA-induced platelet aggregation, ATP release, calcium mobilization, and surface P-selectin expression. Washed human platelets (3.6 × 10^8^ cells/mL) were pretreated with 0.1% DMSO or various concentrations of eugenol (1–5 μM) for 3 min. Subsequently, AA (60 μM) was added to induce (**A**) platelet aggregation, (**B**) ATP release, and (**C**) relative [Ca^2+^]i level. Statistical analyses are presented in the right panels of each subfigure. Data are presented as means ± standard error of the mean (*n* = 4). * *p* < 0.05, and *** *p* < 0.001 indicate differences between the 0.1% DMSO and eugenol groups.

**Figure 2:**
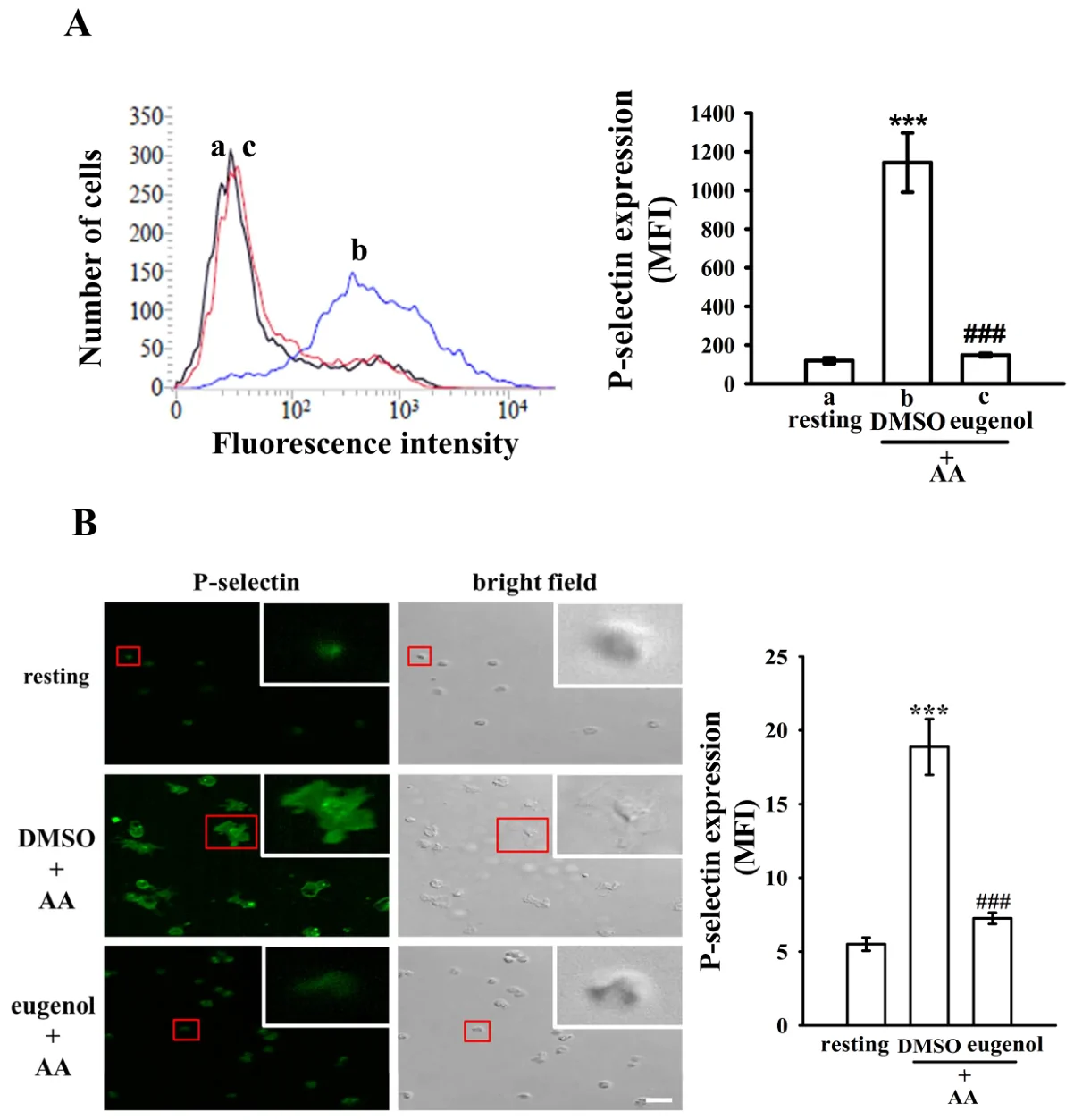
Eugenol suppresses AA-induced P-selectin exposure on the platelet surface. Washed human platelets were treated with 0.1% DMSO or eugenol (5 μM) for 3 min, followed by stimulation with AA (60 μM) to induce platelet activation. (**A**) Flow cytometric analysis of surface P-selectin expression, expressed as mean fluorescence intensity (MFI), in the following groups: (a) resting platelets; (b) AA-stimulated platelets treated with 0.1% DMSO; and (c) AA-stimulated platelets treated with eugenol. (**B**) Representative confocal laser fluorescence microscopy images showing surface P-selectin expression in platelets stained with FITC-conjugated anti-P-selectin antibody (green). Data are presented as means ± standard error of the mean (*n* = 4). *** *p* < 0.001 compared with resting platelets; ^###^ *p* < 0.001 compared with 0.1% DMSO group. The scale bar is 5 μm.

Eugenol (1–5 μM) concentration-dependently inhibited AA (60 μM)-induced platelet aggregation in washed human platelets, with an approximate IC_50_ of 2.5 μM (Figure 1A). Since 5 μM produced the maximal inhibitory effect within the tested concentration range, this concentration was used for subsequent in vitro experiments. Platelet activation is closely linked to the release of granular contents, including ATP and Ca^2+^, which amplify platelet aggregation and activation. As illustrated in Figure 1B,C, eugenol (5 μM) significantly suppressed both the ATP release (94% reduction) and the mobilization of cytosolic calcium (83.4% reduction) triggered by AA. P-selectin, a key marker of platelet activation, is normally located on the inner surface of α-granules but exposed on the platelet surface upon activation [23]. Eugenol (5 μM) significantly reduced AA-induced P-selectin expression (a, resting, 118 ± 15; b, 0.1% dimethyl sulfoxide (DMSO) + AA group, 1144 ± 153; c, 5 µM eugenol + AA group, 148 ± 9; Figure 2A), as observed by flow cytometric analysis. This inhibitory effect of eugenol on P-selectin surface expression was further detected through confocal laser fluorescence microscopy, which showed consistent results (Figure 2B, green fluorescence). These results indicate that eugenol inhibits the granule secretion processes involved in AA-induced platelet activation.

### 3.2. Regulation of MAPK Activation by Eugenol in Platelet Activation

**Figure 3:**
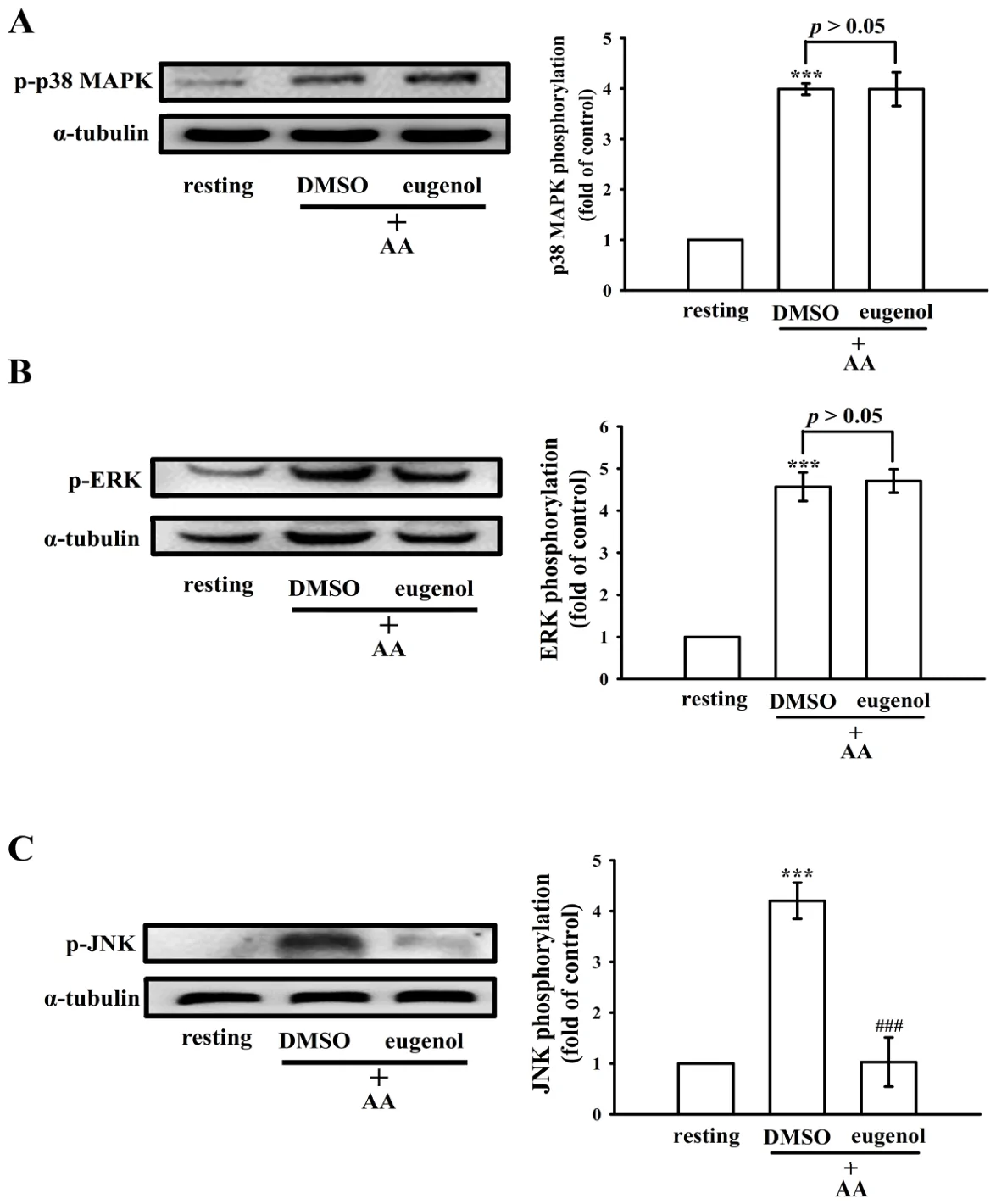
Effects of eugenol on MAPK phosphorylation in platelets. Washed human platelets (3.6 × 10^8^ cells/mL) were incubated with either 0.1% DMSO or eugenol (5 µM) for 3 min and subsequently exposed to AA (60 μM). The phosphorylation levels of MAPK family members, including (**A**) p38 MAPK, (**B**) ERK, and (**C**) JNK, were examined by immunoblotting. Data are presented as means ± standard error of the mean (*n* = 4). *** *p* < 0.001 compared with resting platelets; ^###^ *p* < 0.001 compared with 0.1% DMSO group.

**Figure 4:**
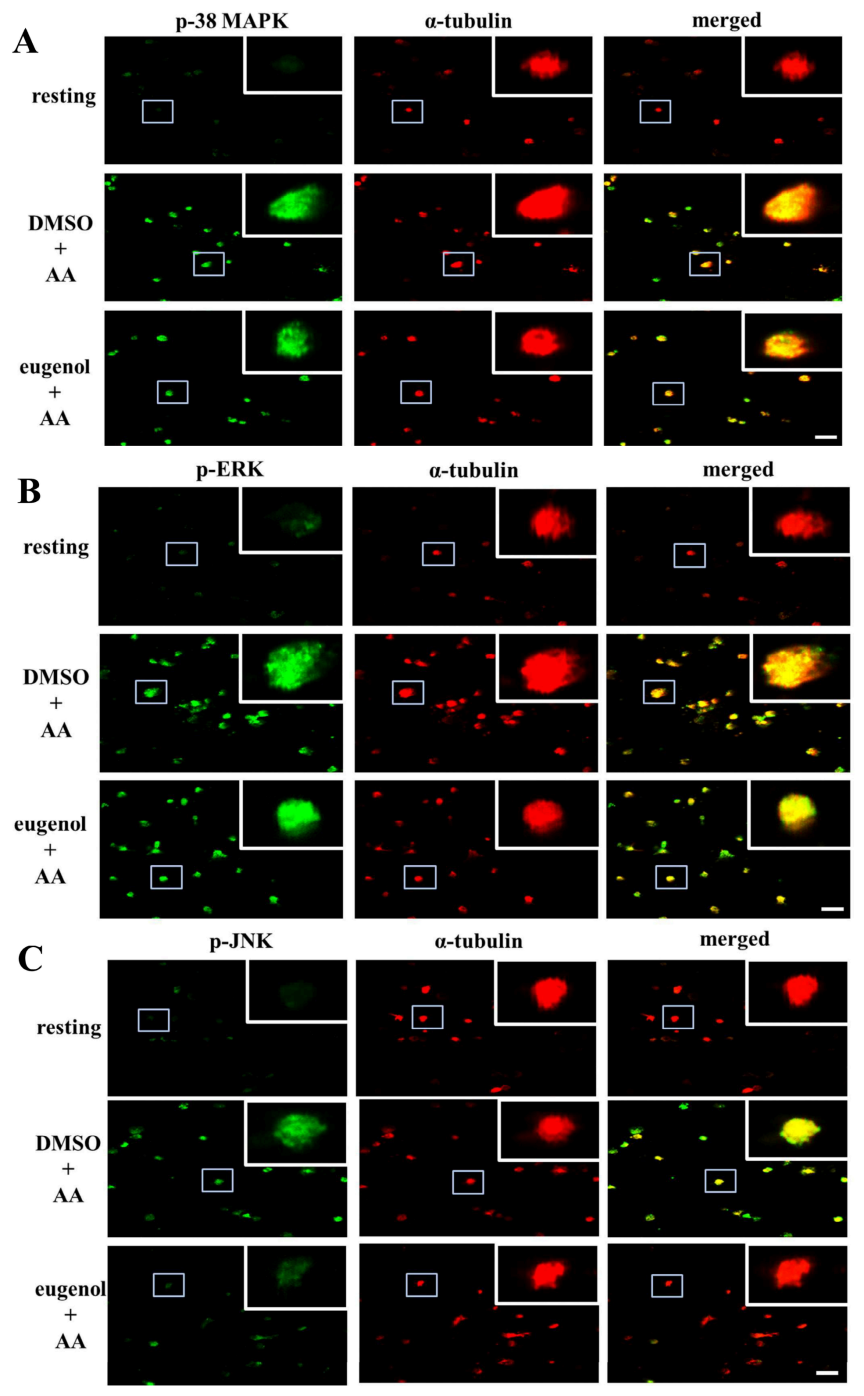
Eugenol-induced inhibition of MAPK activation on confocal laser microscopy. Washed platelets (3.6 × 10^8^ cells/mL) were preincubated with 0.1% DMSO or eugenol (5 µM) for 3 min and subsequently exposed to AA (60 μM) for confocal microscopic evaluation at 1000× magnification, focusing on visualization of (**A**) phosphorylated p38 MAPK, (**B**) ERK, and (**C**) JNK through green fluorescence and α-tubulin through red fluorescence. Images represent four independent experiments. The scale bar is 5 μm.

In the MAPK signaling pathways activated by AA, p38 phosphorylation initiates a cascade that promotes granule secretion and platelet aggregation [24,25]. ERK activation regulates AA release in platelets, and JNK plays a major role in TXA_2_ generation [26]. Therefore, we examined MAPK phosphorylation in AA-stimulated platelet activation and the effect of eugenol on these pathways. AA induced phosphorylation in all three MAPKs (Figure 3), whereas eugenol (5 μM) selectively inhibited JNK phosphorylation (*p* = 2.4 × 10^−4^ vs. DMSO + AA; Figure 3C; *p* = 3.2 × 10^−4^; η^2^ = 0.862). In the confocal fluorescence microscopy images (Figure 4), green fluorescence (MAPK phosphorylation) and blue fluorescence (α-tubulin) were observed in both resting and AA-activated platelets (Figure 4). AA-activated platelets exhibited increased fluorescence for phosphorylated p38 MAPK, ERK, and JNK relative to resting platelets. However, eugenol treatment (5 µM) only reduced JNK phosphorylation, and no apparent changes in α-tubulin fluorescence were observed among the experimental groups. Collectively, these results indicate that eugenol specifically inhibits JNK phosphorylation to modulate AA-induced platelet activation.

### 3.3. Inhibition of Platelet Aggregation, ATP Release, Cytosolic Calcium Mobilization, and P-Selectin Expression by SP600125 in AA-Stimulated Platelet Activation

**Figure 5:**
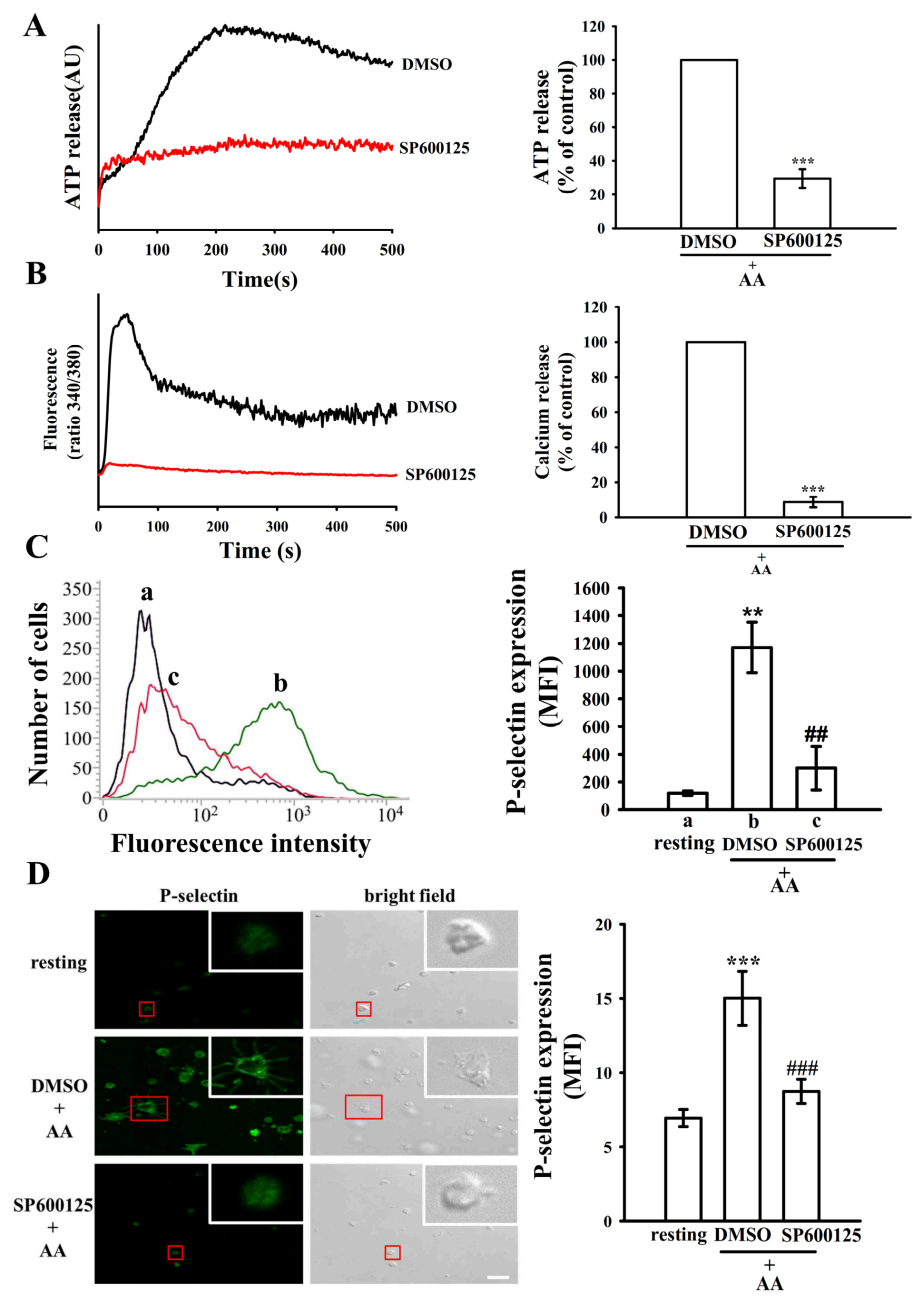
SP600125-induced regulation of ATP release, calcium mobilization, and surface P-selectin expression in AA-induced platelet activation. Washed human platelets (3.6 × 10^8^ cells/mL) were preincubated with 0.1% DMSO or SP600125 (20 μM) for 3 min. Subsequently, AA (60 μM) was added to trigger (**A**) ATP release, (**B**) calcium mobilization, and (**C**) Surface P-selectin expression, presented as MFI: (a) resting; (b) 0.1% DMSO + AA; (c) SP600125 + AA. (**D**) Representative confocal laser fluorescence microscopy images of platelets stained with FITC-conjugated anti-P-selectin antibody (green fluorescence) to visualize surface P-selectin expression. Data are expressed as means ± standard error of the mean (*n* = 4). In (**A**,**B**), *** *p* < 0.001 indicates differences between the SP600125- and 0.1% DMSO-treated groups. In (**C**,**D**), ** *p* < 0.01 and *** *p* < 0.001 indicate differences between the resting and 0.1% DMSO-treated groups, and ^##^ *p* < 0.01 and ^###^ *p* < 0.001 indicate differences between the SP600125- and 0.1% DMSO-treated groups. The scale bar is 5 μm.

JNK regulates α-granule P-selectin expression and δ-granule ATP release in response to collagen, thrombin, and ADP [27]. Eugenol selectively inhibited JNK phosphorylation (Figure 3), thereby modulating AA-induced platelet activation. This finding suggests that JNK may play a key role in AA-induced platelet activation. Thus, we evaluated the effects of the JNK inhibitor SP600125 on platelet aggregation, ATP release, cytosolic calcium mobilization, and P-selectin expression in AA-stimulated platelets. SP600125 (20 μM) markedly inhibited AA-induced platelet aggregation compared with the 0.1% DMSO control group (Figure S1). It also decreased ATP release (Figure 5A), cytosolic calcium mobilization (Figure 5B), and surface P-selectin expression (a, resting, 120 ± 16; b, 0.1% DMSO + AA group, 1171 ± 182; c, 20 µM SP600125 + AA group, 300 ± 157; Figure 5C). Consistent with the flow cytometric analysis, confocal imaging results also showed that SP600125 significantly suppressed AA-induced surface P-selectin expression (Figure 5D). These findings indicate that JNK contributes to AA-stimulated platelet responses, particularly by regulating granule secretion and cytosolic calcium mobilization.

### 3.4. Role of Intracellular Cyclic Nucleotides in Antiplatelet Activity of Eugenol

**Figure 6:**
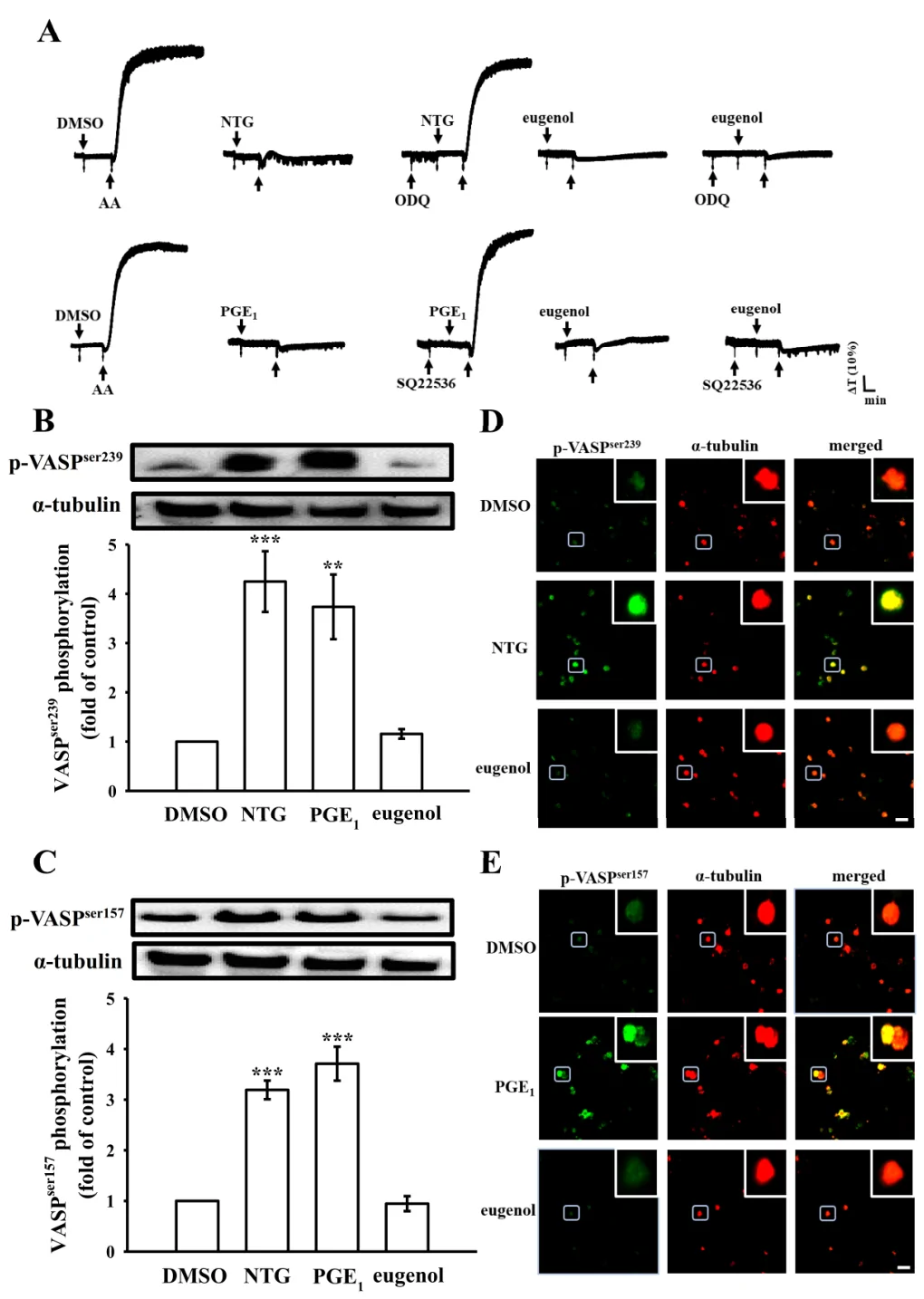
Cyclic nucleotide/VASP signaling is not involved in the antiplatelet action of eugenol. (**A**) Platelet aggregation was assessed in washed human platelets (3.6 × 10^8^ cells/mL) after a 3 min treatment with NTG (5 µM), PGE1 (10 nM), or eugenol (5 µM) in the absence or presence of the guanylate cyclase inhibitor ODQ (10 µM) or the adenylyl cyclase inhibitor SQ22536 (10 µM). Aggregation was initiated by AA (60 µM). In parallel experiments, platelets were treated with 0.1% DMSO, NTG (5 µM), PGE1 (10 nM), or eugenol (5 µM), and the phosphorylation of (**B**) VASP Ser^239^ and (**C**) VASP Ser^157^ was determined by immunoblot analysis. Immunofluorescence staining was further performed to detect phosphorylated (**D**) VASP Ser^239^ and (**E**) VASP Ser^157^ in green, with α-tubulin counterstained in red. Data from four independent experiments are shown for (**A**,**D**,**E**). Bar graphs are expressed as the mean ± standard error of the mean (*n* = 4). ** *p* < 0.01 and *** *p* < 0.001 compared with the 0.1% DMSO-treated group. Scale bar = 5 µm.

Both NTG and PGE_1_ elevate intracellular levels of the cyclic nucleotides, cGMP and cAMP, which promote VASP phosphorylation and inhibit platelet activation, producing antiplatelet effects. To investigate whether cyclic nucleotide signaling is involved in eugenol-mediated platelet inhibition, we used ODQ, a guanylate cyclase inhibitor, to block cGMP production. SQ22536, an adenylate cyclase inhibitor, was used to suppress cAMP synthesis. As displayed in Figure 6A, ODQ (10 μM) and SQ22536 (10 μM) significantly counteracted the inhibitory effects of NTG (5 μM) and PGE_1_ (10 nM) on AA-stimulated platelet aggregation. However, neither ODQ nor SQ22536 significantly affected the antiplatelet activity induced by eugenol (5 μM). VASP phosphorylation differed significantly among groups at Ser239 (*p* = 2.2 × 10^−4^, η^2^ = 0.779) and Ser157 (*p* = 2.1 × 10^−7^, η^2^ = 0.926). NTG (5 μM; 95% CI, 1.6–4.8; *p* = 7.9 × 10^−4^ vs. DMSO) and PGE_1_ (10 nM; 95% CI, 1.3–4.2; *p* = 0.0014 vs. DMSO) significantly increased VASP Ser239 phosphorylation. Similar increases were observed in VASP Ser157 phosphorylation following treatment with NTG (95% CI, 1.8–2.6; *p* = 1.4 × 10^−6^ vs. DMSO) and PGE_1_ (95% CI, 2.2–3.3; *p* = 6.0 × 10^−7^ vs. DMSO). In contrast, eugenol (5 μM) did not significantly affect VASP phosphorylation at either Ser239 (95% CI, −1.0–1.3; *p* = 0.770 vs. DMSO) or Ser157 (95% CI, −0.4–0.5; *p* = 0.791 vs. DMSO; Figure 6B,C). On confocal fluorescence microscopy images (Figure 6D,E), green fluorescence (VASP phosphorylation) and red fluorescence (α-tubulin) in platelets further indicated the inhibitory effect induced by NTG, PGE_1_ and eugenol. NTG and PGE1 markedly increased the fluorescence intensity of phosphorylated VASP compared with resting platelets, whereas eugenol did not enhance VASP phosphorylation. No apparent difference in α-tubulin fluorescence intensity was observed among the groups (Figure 6D,E). These results indicate that the inhibitory effect of eugenol on platelet aggregation is not associated with increased intracellular cyclic nucleotide signaling.

### 3.5. In Vivo Evaluation of Eugenol-Induced Antithrombotic Activity

**Figure 7:**
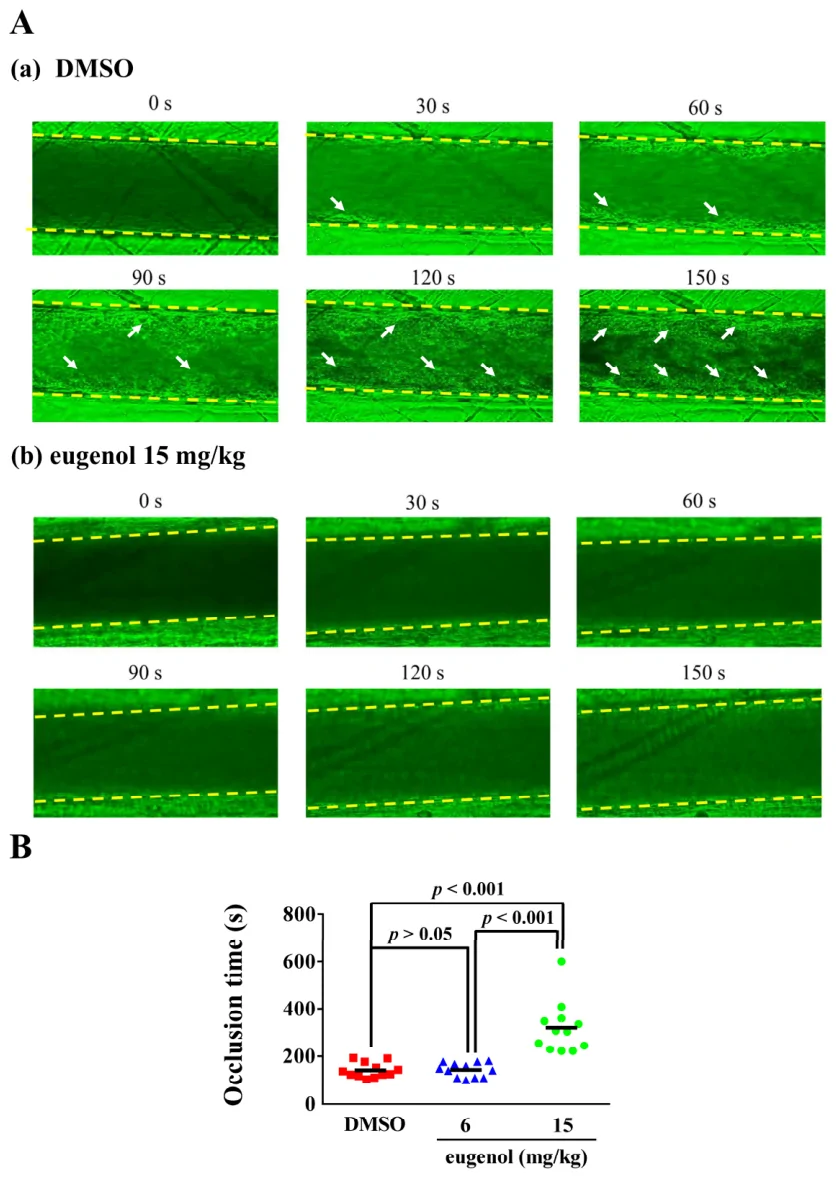
Efficacy of eugenol in platelet plug occlusion in mesenteric vessels. Mice received intraperitoneal administration of either a solvent control (0.1% DMSO) or eugenol at doses of 6 and 15 mg/kg, and mesenteric vessels were subjected to fluorescein irradiation to induce thrombus formation. In panel (**A**), real-time microscopic images were captured at intervals from 0 to 150 s post-irradiation at 400× magnification. Yellow dashed lines and white arrows indicate the blood vessel wall and thrombus formation. In panel (**B**), occlusion time was quantified as the primary measure, and data are presented as means ± standard error of the mean (*n* = 12).

**Figure 8:**
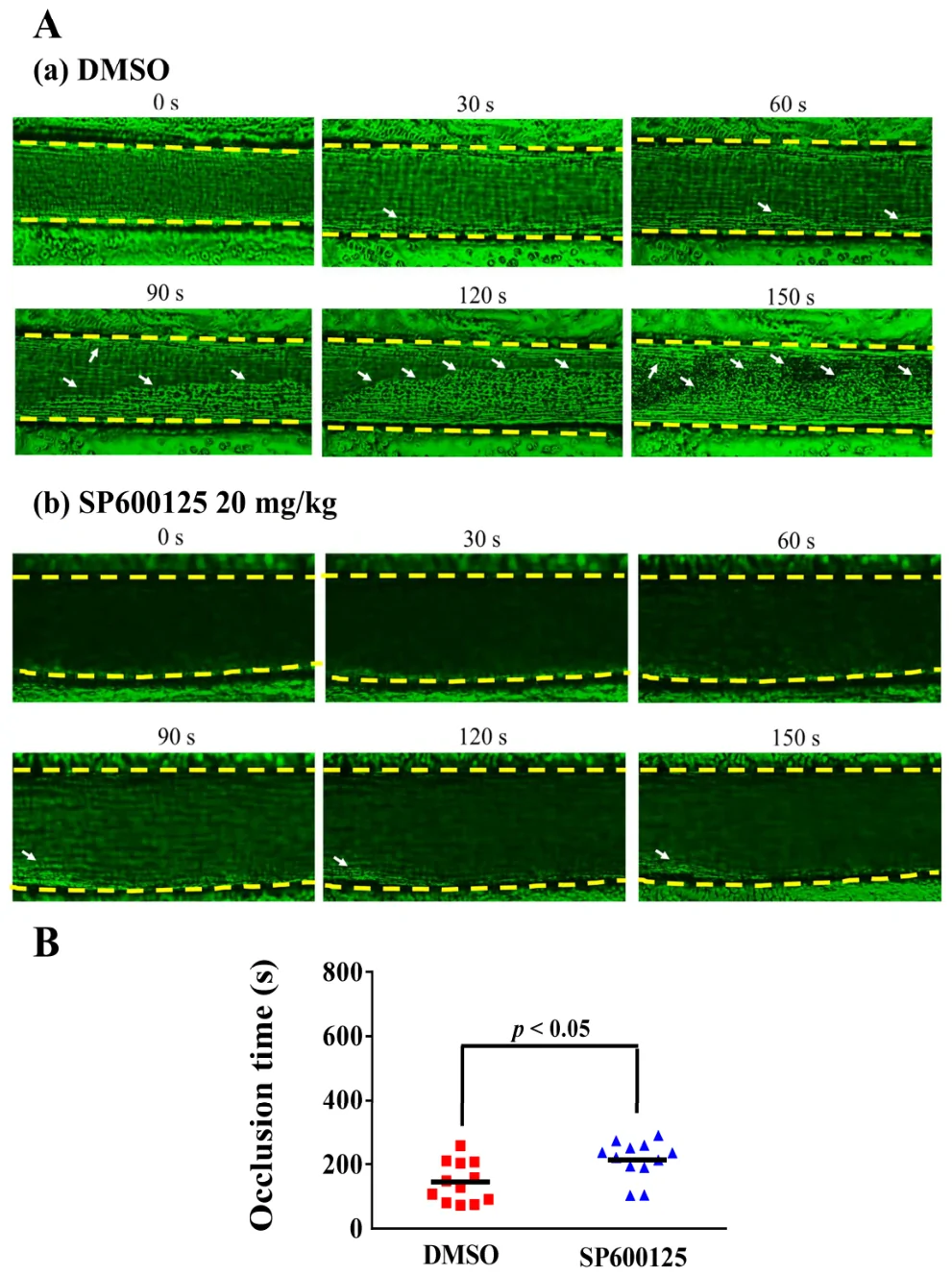
Efficacy of SP600125 in platelet plug occlusion in mesenteric vessels. Mice received intraperitoneal administration of either a solvent control (0.1% DMSO) or SP600125 at doses of 20 mg/kg, and mesenteric vessels were subjected to fluorescein irradiation to induce thrombus formation. In panel (**A**), real-time microscopic images were captured at intervals from 0 to 150 s post-irradiation at 400× magnification. Yellow dashed lines and white arrows indicate the blood vessel wall and thrombus formation. In panel (**B**), occlusion time was quantified as the primary measure, and data are presented as means ± standard error of the mean (*n* = 12).

A previous study reported that eugenol administration at 6 or 15 mg/kg prevented platelet plug formation in microvessels [28]. We extended this investigation to the antithrombotic activity of eugenol by examining the time course of fluorescein-induced platelet plug formation in the mesenteric arteries of mice (Figure 7A). In the 0.1% DMSO group, discrete platelet aggregates formed along the vessel wall at 30 s post-irradiation. By 60 s, partial vessel occlusion was evident. Progressive thrombus expansion markedly reduced blood flow by 90 s, and this effect was further exacerbated by 120 s. Complete occlusion was observed at 150 s, confirming the rapid progression of thrombus formation. However, mice treated with 15 mg/kg eugenol exhibited no discernible platelet aggregation at 30 or 60 s. Thrombus formation remained negligible at 90 s, and the vessel remained patent at 150 s, suggesting eugenol has potent antithrombotic effects (Figure 7A). These observations were quantified by measuring the occlusion time (Figure 7B; *p* = 7.0 × 10^−8^, η^2^ = 0.632). Treatment with 6 mg/kg eugenol did not significantly alter occlusion time compared with 0.1% DMSO (143 ± 9 s vs. 141 ± 9 s; 95% CI, −53.7–57.2; *n* = 12 per group; *p* = 0.9492). By contrast, treatment with 15 mg/kg eugenol significantly prolonged the occlusion time compared with 0.1% DMSO (319 ± 31 s vs. 141 ± 9 s; 95% CI, 111.4–245.1; *n* = 12 per group; *p* = 5.8 × 10^−7^). A direct comparison further showed that the occlusion time was significantly longer in the 15 mg/kg eugenol group than in the 6 mg/kg group (95% CI, 121.1–231.9; *p* = 2.4 × 10^−7^). To further investigate the involvement of JNK in thrombus formation in vivo, mice were treated with the JNK inhibitor SP600125 in the same experimental model. SP600125 (20 mg/kg) significantly prolonged occlusion time compared with 0.1% DMSO (214 ± 17 s vs. 145 ± 18 s; 95% CI, 17.5–120.7; *n* = 12 per group; *p* = 0.011; η^2^ = 0.259; Figure 8).

## 4. Discussion

**Figure 9:**
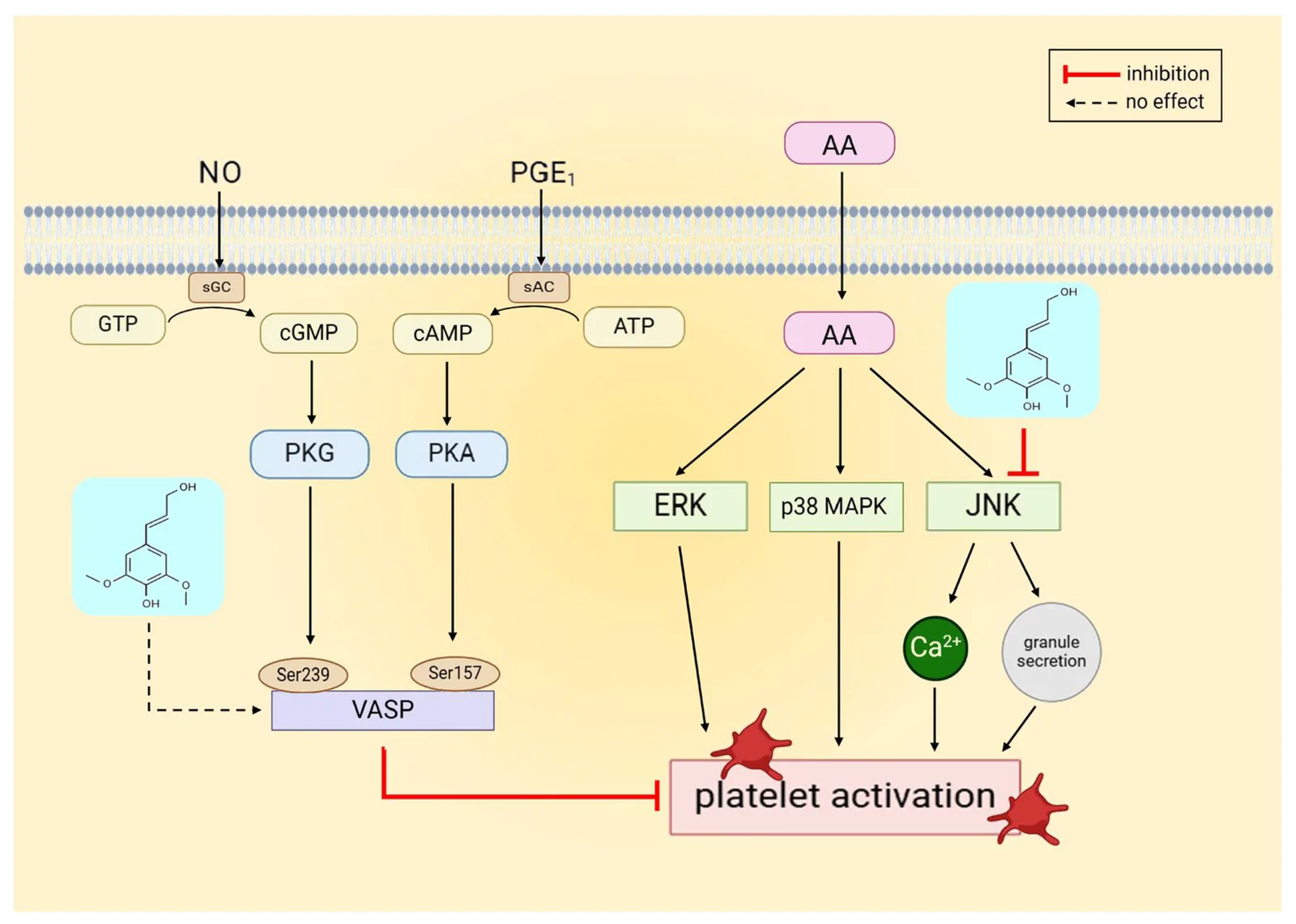
Potential mechanisms through which eugenol inhibits AA-induced platelet activation. Eugenol inhibits AA-induced platelet activation, which may involve modulation of JNK phosphorylation. Under therapeutically relevant conditions, eugenol does not affect NO/cGMP or PGE1/cAMP signaling, as indicated by unchanged VASP phosphorylation levels. GTP: guanosine triphosphate, NO: nitric oxide, PKA: protein kinase A (cAMP-dependent), PKG: protein kinase G (cGMP-dependent), sGC: soluble guanylate cyclase, sAC: soluble adenylate cyclase.

The present study shows that the antiplatelet and antithrombotic effects of eugenol are associated with attenuation of JNK signaling rather than activation of the cyclic nucleotide/VASP pathway, as summarized in Figure 9. These findings extend our previous study showing that eugenol inhibits collagen- and AA-induced platelet activation and attenuates AA-induced cPLA_2_ phosphorylation and TXB_2_ formation [19]. In the present study, SP600125 also reduced AA-induced TXB_2_ formation (Figure S2), suggesting that JNK may contribute to AA-dependent TXA_2_ generation. Thus, attenuation of JNK phosphorylation may partly contribute to the inhibitory effect of eugenol on the AA–TXA_2_ pathway. Given that AA activates platelets primarily via the thromboxane receptor and collagen via the glycoprotein VI (GPVI) receptor [29], the ability of eugenol to inhibit responses to both agonists suggests it does not act through a specific membrane receptor. These findings support the hypothesis that eugenol may act within the cytoplasm and involve modulation of JNK phosphorylation, rather than targeting platelet receptors. This mechanism highlights the potential of eugenol as a novel antithrombotic agent for the regulation of platelet function in thrombotic disorders.

MAPK family members involved in platelet signaling include ERK1/2, p38 MAPK, JNK, and ERK5. While ERK5 has been implicated in platelet responses under oxidative or metabolic stress, previous studies have demonstrated that it is not activated by either AA or collagen stimulation in platelets [27,28]. In contrast, AA stimulation induces the phosphorylation of JNK, ERK1/2, and p38 MAPK. Among these classical MAPKs, ERK1/2 mainly regulates cytoskeletal rearrangement and granule secretion, particularly downstream of collagen–GPVI signaling [27,30], whereas p38 MAPK contributes to TxA_2_ synthesis through cPLA_2_ activation, especially following thrombin or collagen stimulation [31]. JNK has broader roles in integrin α_IIb_β_3_ activation and secretion induced by multiple agonists, including thrombin, ADP, and collagen [25]. Moreover, JNK-deficient platelets exhibit reduced collagen-induced TxA_2_ generation [27], indicating its involvement in TxA_2_ synthesis. In the present study, eugenol selectively inhibited AA-induced JNK phosphorylation without affecting ERK1/2 or p38 MAPK. Consistently, the JNK inhibitor SP600125 markedly suppressed AA-induced platelet aggregation, supporting a specific role for JNK in this response.

VASP plays an important role in regulating platelet responsiveness and serves as a major downstream effector of cyclic nucleotide signaling. In platelets, increases in intracellular cAMP and cGMP activate PKA and PKG, respectively, leading to VASP phosphorylation at Ser157 and Ser239 [32]. Phosphorylated VASP contributes to the suppression of platelet activation by interfering with cytoskeletal rearrangement, integrin activation, calcium mobilization, and PLC/PKC-related signaling events [11,33]. This inhibitory mechanism has also been linked to reduced platelet reactivity and attenuation of AA–TXA_2_-mediated proaggregatory signaling [34,35]. Nevertheless, our data showed that blockade of adenylate cyclase by SQ22536 or guanylate cyclase by ODQ did not restore AA-induced platelet aggregation in the presence of eugenol. Moreover, eugenol treatment did not produce a significant change in VASP phosphorylation at either Ser157 or Ser239. These observations indicate that eugenol suppresses AA-induced platelet activation through a mechanism that is unlikely to depend on the cyclic nucleotide/VASP signaling axis.

Building upon prior studies on microvascular thrombosis [28], we employed a mesenteric artery model to mimic clinical conditions and evaluate the antithrombotic effects of eugenol through a real-time time-course approach. Notably, Huang et al. [19] reported that eugenol did not increase bleeding risk even at 15 mg/kg, in contrast to aspirin—a widely used antiplatelet agent for cardiovascular disease prevention—which is known to significantly prolong bleeding time. These findings support the potential of eugenol as a safer and effective alternative for the prevention of arterial thrombosis. The anatomical and hemodynamic characteristics of the mesenteric artery model increase its clinical relevance as a thrombosis model. These findings suggest that eugenol may effectively modulate platelet function and prevent thrombosis. In addition, the JNK inhibitor SP600125 also significantly prolonged occlusion time in the same in vivo thrombosis model, supporting a role for JNK in thrombus formation.

Several limitations remain in the present study. First, human platelet experiments were conducted using samples from healthy donors aged 20–35 years, without stratification by sex. Although eugenol inhibited platelet activation in both male and female samples, no formal sex-based analysis was performed. As this was a mechanistic study, we prioritized the inclusion of healthy individuals without applying sex-based selection criteria. Second, while we employed SP600125 as a JNK inhibitor, the lack of platelet-specific JNK knockout models and the anucleate nature of platelets preclude the use of genetic manipulation or overexpression strategies. Consequently, pharmacological inhibition remains the most feasible and widely accepted approach in this context. Third, the in vivo thrombosis model used intraperitoneal administration to ensure rapid systemic absorption, which is appropriate for acute efficacy assessment but does not reflect long-term effects. Lastly, the potential effects of eugenol on other hemostatic pathways, including coagulation and fibrinolysis, cannot be excluded and warrant further investigation.

## 5. Conclusions

The present study provides new insights into the regulatory effects of eugenol on human platelet activation. Eugenol effectively suppressed AA-induced platelet activation, and this effect was associated with reduced JNK phosphorylation while remaining independent of the cyclic nucleotide/VASP signaling pathway. Nevertheless, the involvement of additional, unidentified mechanisms in eugenol-mediated platelet inhibition cannot be excluded. These findings suggest that eugenol may serve as a promising antithrombotic candidate for further investigation in thrombotic disorders.

## Data Availability

The original contributions presented in this study are included in the article/Supplementary Material. Further inquiries can be directed to the corresponding authors.

## Supplementary Materials

The following supporting information can be downloaded at: https://www.mdpi.com/article/10.3390/biomedicines14081800/s1, Figure S1: Effects of SP600125 on AA-induced platelet aggregation. Figure S2: Effects of SP600125 on AA-induced thromboxane B_2_ (TxB_2_) formation.

## References

[1] Scridon A. (2022). Platelets and their role in hemostasis and thrombosis-from physiology to pathophysiology and therapeutic implications. Int. J. Mol. Sci..

[2] Lin W., Wang S., Liu R., Zhang D., Zhang J., Qi X., Li Z., Miao M., Cai X., Su G. (2025). Research progress of cPLA2 in cardiovascular diseases (Review). Mol. Med. Rep..

[3] Badimon L., Vilahur G., Rocca B., Patrono C. (2021). The key contribution of platelet and vascular arachidonic acid metabolism to the pathophysiology of atherothrombosis. Cardiovasc. Res..

[4] Zhou Y., Khan H., Xiao J., Cheang W.S. (2021). Effects of arachidonic acid metabolites on cardiovascular health and disease. Int. J. Mol. Sci..

[5] Yue J., López J.M. (2020). Understanding MAPK signaling pathways in apoptosis. Int. J. Mol. Sci..

[6] Cargnello M., Roux P.P. (2011). Activation and function of the MAPKs and their substrates, the MAPK-activated protein kinases. Microbiol. Mol. Biol. Rev..

[7] Nakashima S., Chatani Y., Nakamura M., Miyoshi N., Kohno M., Nozawa Y. (1994). Tyrosine phosphorylation and activation of mitogen-activated protein kinases by thrombin in human platelets: Possible involvement in late arachidonic acid release. Biochem. Biophys. Res. Commun..

[8] Flevaris P., Li Z., Zhang G., Zheng Y., Liu J., Du X. (2009). Two distinct roles of mitogen-activated protein kinases in platelets and a novel Rac1-MAPK-dependent integrin outside-in retractile signaling pathway. Blood.

[9] Fan X., Wang C., Shi P., Gao W., Gu J., Geng Y., Yang W., Wu N., Wang Y., Xu Y. (2018). Platelet MEKK3 regulates arterial thrombosis and myocardial infarct expansion in mice. Blood Adv..

[10] Li Z., Ajdic J., Eigenthaler M., Du X. (2003). A predominant role for cAMP-dependent protein kinase in the cGMP-induced phosphorylation of vasodilator-stimulated phosphoprotein and platelet inhibition in humans. Blood.

[11] Benz P.M., Frömel T., Laban H., Zink J., Ulrich L., Groneberg D., Boon R.A., Poley P., Renne T., de Wit C. (2023). Cardiovascular functions of Ena/VASP proteins: Past, present and beyond. Cells.

[12] Darius H., Michael-Hepp J., Thierauch K.H., Fisch A. (1994). Inhibition of human platelets and polymorphonuclear neutrophils by the potent and metabolically stable prostaglandin D2 analog ZK 118.182. Eur. J. Pharmacol..

[13] Stanger L., Yamaguchi A., Holinstat M. (2023). Antiplatelet strategies: Past, present, and future. J. Thromb. Haemost..

[14] Grove E.L., Würtz M., Schwarz P., Jørgensen N.R., Vestergaard P. (2013). Gastrointestinal events with clopidogrel: A nationwide population-based cohort study. J. Gen. Intern. Med..

[15] Lao Y., Guo J., Fang J., Geng R., Li M., Qin Y., Wu J., Kang S.G., Huang K., Tong T. (2024). Beyond flavor: The versatile roles of eugenol in health and disease. Food Funct..

[16] Hui Q., Ammeter E., Liu S., Yang R., Lu P., Lahaye L., Yang C. (2020). Eugenol attenuates inflammatory response and enhances barrier function during lipopolysaccharide-induced inflammation in the porcine intestinal epithelial cells. J. Anim. Sci..

[17] Hwang S.M., Lee K., Im S.T., Go E.J., Kim Y.H., Park C.K. (2020). Co-application of eugenol and QX-314 elicits the prolonged blockade of voltage-gated sodium channels in nociceptive trigeminal ganglion neurons. Biomolecules.

[18] Devi K.P., Nisha S.A., Sakthivel R., Pandian S.K. (2010). Eugenol (an essential oil of clove) acts as an antibacterial agent against *Salmonella typhi* by disrupting the cellular membrane. J. Ethnopharmacol..

[19] Huang W.C., Shu L.H., Kuo Y.J., Lai K.S., Hsia C.W., Yen T.L., Hsia C.H., Jayakumar T., Yang C.H., Sheu J.R. (2024). Eugenol suppresses platelet activation and mitigates pulmonary thromboembolism in humans and murine models. Int. J. Mol. Sci..

[20] Hsia C.W., Shu L.H., Lee A.W., Tran O.T., Yang C.H., Yen T.L., Huang W.C., Hsia C.H., Jayakumar T., Chiou K.R. (2024). Ginkgetin effectively mitigates collagen and AA-induced platelet activation via PLCγ2 but not cyclic nucleotide-dependent pathway in human. J. Cell. Mol. Med..

[21] Hsia C.W., Huang W.C., Jayakumar T., Hsia C.H., Hou S.M., Chang C.C., Yen T.L., Sheu J.R. (2023). Garcinol acts as a novel integrin αIIbβ3 inhibitor in human platelets. Life Sci..

[22] Lu W.J., Tsai C.H., Chen R.J., Huang L.T., Chen T.Y., Chen L.C., Chen H.H., Wang H.H., Peng H.Y., Sun Y.Y. (2022). Artesunate as a glycoprotein VI antagonist for preventing platelet activation and thrombus formation. Biomed. Pharmacother..

[23] Ambrosio A.L., Di Pietro S.M. (2025). The winding road to platelet α-granules. Front. Cell Dev. Biol..

[24] Barry O.P., Kazanietz M.G., Praticò D., FitzGerald G.A. (1999). Arachidonic acid in platelet microparticles up-regulates cyclooxygenase-2-dependent prostaglandin formation via a protein kinase C/mitogen-activated protein kinase-dependent pathway. J. Biol. Chem..

[25] Adam F., Kauskot A., Rosa J.P., Bryckaert M. (2008). Mitogen-activated protein kinases in hemostasis and thrombosis. J. Thromb. Haemost..

[26] Patel P., Naik U.P. (2020). Platelet MAPKs-a 20+ year history: What do we really know?. J. Thromb. Haemost..

[27] Adam F., Kauskot A., Nurden P., Sulpice E., Hoylaerts M.F., Davis R.J., Rosa J.P., Bryckaert M. (2010). Platelet JNK1 is involved in secretion and thrombus formation. Blood.

[28] Chang Y., Hsia C.W., Chiou K.R., Yen T.L., Jayakumar T., Sheu J.R., Huang W.C. (2024). Eugenol: A potential modulator of human platelet activation and mouse mesenteric vascular thrombosis via an innovative cPLA2-NF-κB signaling axis. Biomedicines.

[29] Estevez B., Du X. (2017). New concepts and mechanisms of platelet activation signaling. Physiology.

[30] Shankar H., Garcia A., Prabhakar J., Kim S., Kunapuli S.P. (2006). P2Y12 receptor-mediated potentiation of thrombin-induced thromboxane A2 generation in platelets occurs through regulation of Erk1/2 activation. J. Thromb. Haemost..

[31] Kramer R.M., Roberts E.F., Um S.L., Börsch-Haubold A.G., Watson S.P., Fisher M.J., Jakubowski J.A. (1996). p38 mitogen-activated protein kinase phosphorylates cytosolic phospholipase A2 (cPLA2) in thrombin-stimulated platelets. Evidence that proline-directed phosphorylation is not required for mobilization of arachidonic acid by cPLA2. J. Biol. Chem..

[32] Benz P.M., Laban H., Zink J., Günther L., Walter U., Gambaryan S., Dib K. (2016). Vasodilator-stimulated phosphoprotein (VASP)-dependent and -independent pathways regulate thrombin-induced activation of Rap1b in platelets. Cell Commun. Signal..

[33] Schwarz U.R., Walter U., Eigenthaler M. (2001). Taming platelets with cyclic nucleotides. Biochem. Pharmacol..

[34] Irfan M., Kim M., Rhee M.H. (2020). Anti-platelet role of Korean ginseng and ginsenosides in cardiovascular diseases. J. Ginseng Res..

[35] Sheu J.-R., Huang W.-C., Chang C.-C., Hsia C.-W., Hsia C.-H., Jayakumar T., Hou S.-M. (2026). Distinct thromboxane A_2_-dependent pathways regulate arachidonic acid-triggered VASP phosphorylation at Ser239 and Ser157 in human platelets: Real-time visualization reveals superior antithrombotic efficacy by targeting thromboxane A_2_ signaling over cyclooxygenase inhibition. J. Cell. Physiol..

