# Supplementary material for: Eugenol Exhibits Cyclic Nucleotide/VASP-Independent Antiplatelet Activity Associated with Inhibition of Arachidonic Acid-Induced JNK Phosphorylation and Reduces Thrombus Formation as Visualized by Real-Time Intravital Imaging
Source: Biomedicines. 2026 Aug 11;14(8):1800. doi: 10.3390/biomedicines14081800 (PMC13510201; doi:10.3390/biomedicines14081800)
Supplement: Supplementary file 1 [file biomedicines-14-01800-s001.zip › Figure S1.pdf]

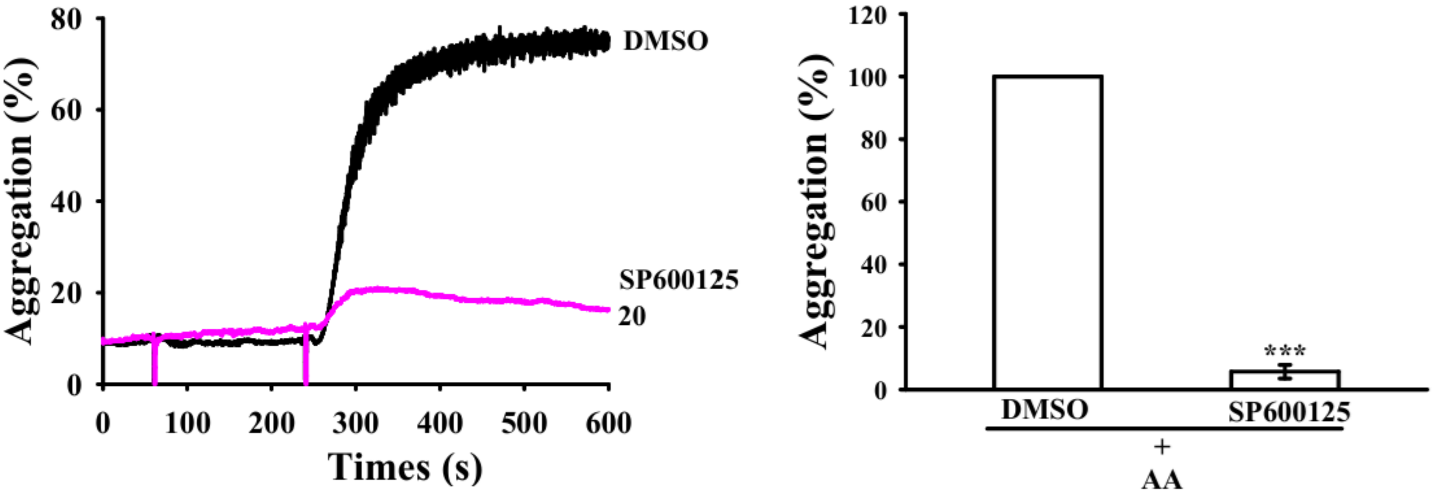

Figure. S1. Effects of SP600125 on AA-induced platelet aggregation. Washed human platelets (3.6×10^8 cells/mL) were pretreated with 0.1% DMSO or SP600125 (20 μM) for 3 min. Subsequently, AA (60 μM) was added to induce platelet aggregation. Data are presented as means ± standard error of the mean ($n = 4$). $^{***}p < 0.001$ indicates differences between the 0.1% DMSO and SP600125 groups.
