# Supplementary material for: Eugenol Exhibits Cyclic Nucleotide/VASP-Independent Antiplatelet Activity Associated with Inhibition of Arachidonic Acid-Induced JNK Phosphorylation and Reduces Thrombus Formation as Visualized by Real-Time Intravital Imaging
Source: Biomedicines. 2026 Aug 11;14(8):1800. doi: 10.3390/biomedicines14081800 (PMC13510201; doi:10.3390/biomedicines14081800)
Supplement: Supplementary file 1 [file biomedicines-14-01800-s001.zip › Figure S2.pdf]

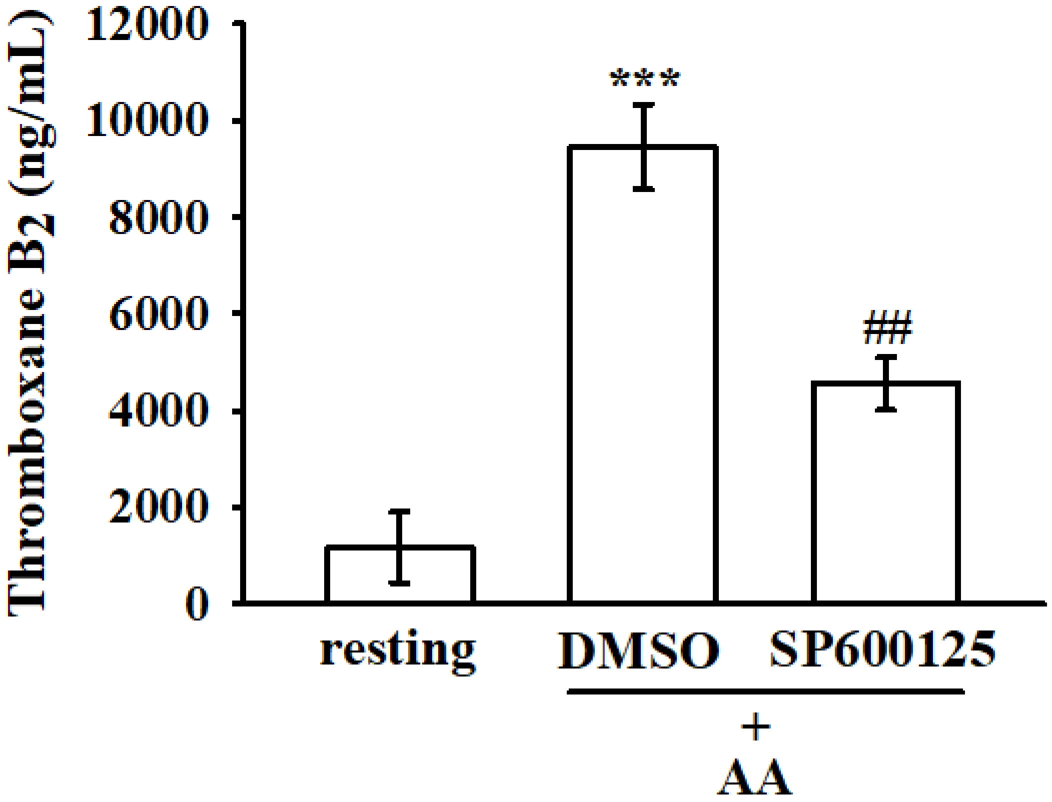

Figure S2. Effects of SP600125 on AA-induced thromboxane B_2 (TxB_2) formation. Washed human platelets were pretreated with 0.1% DMSO or SP600125 (20 μM) for 3 min. Subsequently, AA (60 μM) was added to quantify TxB_2 formation. Data are presented as means ± standard error of the mean ($n = 4$). $^{***}p < 0.001$ indicates differences between the 0.1% DMSO and resting. $^{\#\#}p < 0.01$ indicates differences between the 0.1% DMSO and SP600125 group.
